# Screening Lung Diseases Using Cascaded Feature Generation and Selection Strategies

**DOI:** 10.3390/healthcare10071313

**Published:** 2022-07-14

**Authors:** Jawad Rasheed, Raed M. Shubair

**Affiliations:** 1 Department of Software Engineering, Nisantasi University, Istanbul 34398, Turkey; 2 Department of Electrical and Computer Engineering, New York University (NYU), Abu Dhabi 129188, United Arab Emirates; raed.shubair@nyu.edu

**Keywords:** COVID-19, diagnostic tool, pneumonia, stationary wavelets transformation, transfer learning

## Abstract

The global pandemic COVID-19 is still a cause of a health emergency in several parts of the world. Apart from standard testing techniques to identify positive cases, auxiliary tools based on artificial intelligence can help with the identification and containment of the disease. The need for the development of alternative smart diagnostic tools to combat the COVID-19 pandemic has become more urgent. In this study, a smart auxiliary framework based on machine learning (ML) is proposed; it can help medical practitioners in the identification of COVID-19-affected patients, among others with pneumonia and healthy individuals, and can help in monitoring the status of COVID-19 cases using X-ray images. We investigated the application of transfer-learning (TL) networks and various feature-selection techniques for improving the classification accuracy of ML classifiers. Three different TL networks were tested to generate relevant features from images; these TL networks include AlexNet, ResNet101, and SqueezeNet. The generated relevant features were further refined by applying feature-selection methods that include iterative neighborhood component analysis (iNCA), iterative chi-square (iChi2), and iterative maximum relevance–minimum redundancy (iMRMR). Finally, classification was performed using convolutional neural network (CNN), linear discriminant analysis (LDA), and support vector machine (SVM) classifiers. Moreover, the study exploited stationary wavelet (SW) transform to handle the overfitting problem by decomposing each image in the training set up to three levels. Furthermore, it enhanced the dataset, using various operations as data-augmentation techniques, including random rotation, translation, and shear operations. The analysis revealed that the combination of AlexNet, ResNet101, SqueezeNet, iChi2, and SVM was very effective in the classification of X-ray images, producing a classification accuracy of 99.2%. Similarly, AlexNet, ResNet101, and SqueezeNet, along with iChi2 and the proposed CNN network, yielded 99.0% accuracy. The results showed that the cascaded feature generator and selection strategies significantly affected the performance accuracy of the classifier.

## 1. Introduction

The COVID-19 is a family of viruses that surfaced in China in the last quarter of 2019, and, within a matter of weeks, it affected so many thousands of lives that the World Health Organization (WHO) declared it a pandemic [1]. The devastating effect on the well-being of humankind and the pandemic’s complex non-linear nature has made it difficult to analyze the outbreak [2]. Up to today, more than 560 million COVID-19 positive cases have been detected, while 6.37 million humans have lost their lives. Presently, there are two common methods to diagnose COVID-19: taking samples from the respiratory tract for a kind of viral nucleic acid testing called reverse transcription–polymerase chain reaction (RT-PCR) and analyzing chest radiography imaging. However, RT-PCR requires a sophisticated and specialized laboratory with hi-tech machines that can cost up to USD 90,000 [3]. Besides being expensive, RT-PCR testing has a low positivity rate (63%), with high requirements for procedural and computing time [4]. Contrarily, testing through chest radiography imaging is cheaper, as it does not require the installation of machinery on a large scale due to high availability and easy accessibility in most parts of world [5]. Expert radiologists infer the diagnosis using chest radiography imaging, such as X-ray or computed tomography (CT) imaging, wherein the presence of COVID-19 is inferred by subtle visual signals. This method has gained popularity as a potent alternative. However, it requires additional radiologists, considering the high reproduction number of the COVID-19 virus [6], to analyze the results and reach a conclusion. Thus, to manage and control this pandemic, the immediate and meticulous screening of individuals is of great significance.

In last two decades, ML-based tools have shown promising results in screening and diagnosing several diseases. For instance, authors provided a decision support system to predict diabetes in individuals using various ML classifiers [7]. Similarly, researchers in [8] exploited random forest (RF), k-nearest neighbor (kNN), and naïve Bayes (NB) to detect breast cancer in patients. In addition, deep learning approaches have been successfully deployed in the domain of medical imaging, such as [9], wherein authors developed a TL-based multipurpose diagnostic system to diagnose pediatric pneumonia and some retinal diseases using chest X-ray images and optical coherence tomographic imaging, respectively. Besides ML techniques, computer vision (CV) experts incorporated feature-extraction and image-filtering techniques to enhance system performance in terms of accuracy and computational time. Others, such as [10], investigated principal component analysis (PCA) with a variant of the artificial neural network (ANN) to analyze and identify Parkinson’s disease. Similarly, authors in [11] performed brain tumor classification using wavelet transformation as the feature extractor and SVM as the classifier.

Recently, several artificial-intelligence-based frameworks have been developed to manage the COVID-19 pandemic from different perspectives. Experts in [12] presented a deep-learning-based COVID-19 pneumonia diagnostic tool to distinguish COVID-19 pneumonia from negative cases by utilizing 10,182 chest X-ray radiography images. A textural image-characterization-techniques-based scheme has been proposed in [13] to analyze three ML classifiers (kNN, RF, and SVM) for the identification of COVID-19 positive cases. Their developed scheme achieved 99% accuracy on a test set and 91.3% accuracy on a training set for super-pixel-based histone image characterization. Researchers in [14] classified COVID-19 positive cases by proposing a discrete-wavelet-transform-based neurowavelet capsule network that first minimized the noise present in X-ray images and then performed training for classification. They managed to obtain precision of 99.7%, sensitivity of 99.2%, and accuracy of 99.6%. Another study [15] introduced a deep CNN-based inception model to identify COVID-19 cases by incorporating a Gaussian smoothing filter to enhance image quality and a glowworm swarm optimizer (GSO) for the fine tuning of the hyperparameters. They succeeded in securing 94.29% accuracy and a 93.94% f1-score.

Besides suggesting a single-disease diagnostic tool, computer scientists introduced multi-disease diagnostic systems. For instance, authors in [16] proposed a lung segmentation approach to present a multi-disease diagnostic tool by training a customized CNN-based model to identify tuberculosis, lung opacities, and cancer. Likewise, for the COVID-19 pandemic, researchers in [17] used 6330 images to train 12 well-established pre-trained CNN-based models for discriminating four classes (several pneumonia types including COVID-19). Despite considerable amount of image samples, the system secured an 84.46% average f1-score. A few pre-trained models (Xception, InceptionV3, and ResNeXt) were compared in [18] to identify normal, COVID-19-positive, and pneumonia cases using X-ray images. They obtained an overall accuracy of 86% and a sensitivity of 78%. Another study [19] investigated ResNet50 and VGG16 to classify normal, viral pneumonia and COVID-19 by utilizing 15,153 X-ray images. Their fine-tuned ResNet50 model succeeded in attaining 91.39% accuracy, whereas VGG16 reached the mark of 89.34% accuracy. A more detailed comparison of various studies to manage the COVID-19 pandemic can be found in [1].

**Figure 1:**
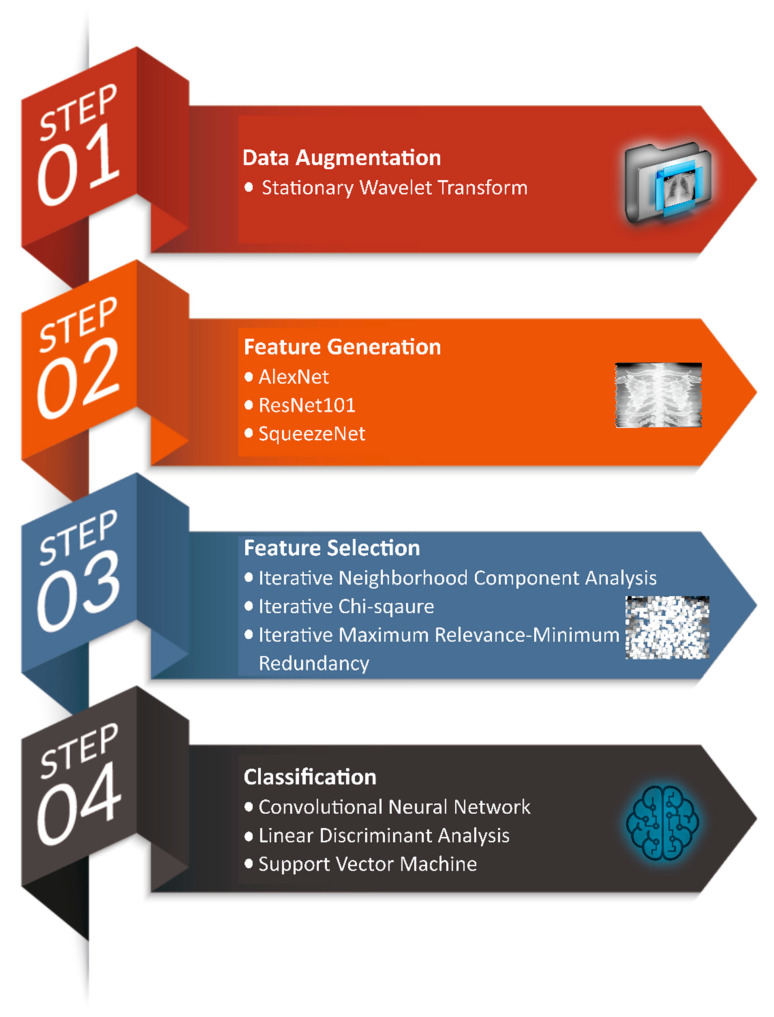
Workflow of the proposed system.

As previously published well-established studies hardly attained effective performance in distinguishing COVID-19 cases from other pneumonia and healthy cases, therefore, to overcome the pandemic, auxiliary methods and techniques for the screening of pneumonia cases need further investigation. In addition to viral nucleic acid testing used in COVID-19 case identification, schemes based on artificial intelligence can be exploited for early diagnosis and monitoring. In this study, we investigated a new framework based on TL networks as a feature generator; several feature-selection techniques; and ML classifiers to identify COVID-19, other (viral/bacterial) pneumonia, and normal cases, using X-ray images with high accuracy (see Figure 1).

In the first phase of this study, X-ray images from three different online sources are gathered, which are then augmented for the better generalization of the proposed model by exploiting stationary wavelet transform and various other techniques, such as the random translation, rotation, and shearing of images. In the second stage, three pre-trained TL models (AlexNet, ResNet101, and SqueezeNet) separately extracted the most-relevant 1000 features from each image. The scheme then merges these features to form a vector of 3000 features. In the third stage, three iterative feature-selection techniques were employed separately to select the most-optimal features for better classification. Lastly, one deep-learning-based framework and two ML classifiers are investigated to perform classification using optimal features.

This study contributes to the research community in the following manner:

- Outlines the prior studies (in detail) related to the identification of COVID-19 and other pneumonia from images.
- Introduces stationary wavelet transform as a data-augmentation technique to handle overfitting issues.
- Investigates the impact of different TL-based networks (AlexNet, ResNet101, and SqueezeNet) in diagnosing three clinical states (normal, other pneumonia, and COVID-19-positive).
- Assesses the significance of incorporating iNCA, iChi2, and iMRMR as feature-selection techniques in ML-based diagnostic tool.
- Analyzes the effect of various combinational schemes of feature-generation and feature-extraction techniques in the proposed model.
- Examines the performance and provides comparative analyses of each combinational scheme in terms of performance accuracy, F1-score, precision, and recall.
- Presents a simple but highly accurate ML-based model that can be used with other conventional clinical COVID-19 testing to remove false-alarm probability.
- Provides analysis to indicate that the proposed scheme outperformed prior studies by securing 99.2% accuracy while segregating COVID-19-positive cases from other pneumonia and normal ones.

The order of the remaining sections of this study is as follows. Section 2 outlines the dataset collection and technical background of the frameworks, techniques, and classifiers used for this study. Section 3 presents the detailed results and performance analysis, while Section 4 illustrates the comparison with previous studies. The paper ends with concluding remarks in Section 5.

## 2. Materials and Methods

The below passages present the overview of data collection and its description, as well as exploratory analysis, collectively with in-depth discussion about the selection and architecture design of pre-trained feature-generation frameworks, feature-selection methods and ML-based disease-identification classifiers adopted for this proposed lung-disease segregation system. In particular, it focuses on data-augmentation techniques, TL models, iterative feature selectors, and ML algorithms used for image data enhancement, the generation of useful features, the automatic selection of optimal features, and disease classification, respectively.

### 2.1. Description of Dataset and Augmentation Approach

The dataset used for this study was created by modifying several publicly available benchmark image repositories to facilitate the evaluation and training of the proposed model. The generated dataset that will be referred as PeN-CoVx throughout this study is a combination of 12,282 images of lungs collected from CRD (COVID-19 Radiography Database|Kaggle: https://www.kaggle.com/tawsifurrahman/covid19-radiography-database) (accessed on 25 March 2022), CIDC (GitHub COVID-19 Image Data Collection: https://github.com/ieee8023/covid-chestxray-dataset) (accessed on 25 March 2022), and CXIP (chest X-ray images (pneumonia)|Kaggle: https://www.kaggle.com/paultimothymooney/chest-xray-pneumonia) (accessed on 25 March 2022). In particular, this research collected the correct data for each class (normal, pneumonia, and COVID-19+) from three different repositories to form PeN-CoVx dataset to avoid overfitting issues. It takes advantage of the below-mentioned types of patient cases:

- 4094 randomly selected X-ray images of normal healthy patients from the CRD dataset,
- 3616 X-ray images of confirmed COVID-19-positive cases from the CRD dataset,
- 478 X-ray images of confirmed COVID-19-positive cases from the CIDC dataset,
- 4094 randomly selected X-ray images of viral and bacterial pneumonia-affected patients from the CXIP dataset.

The idea of easily accessible and open source data inspired the preference of the CRD, CIDC, and CXIP datasets to form PeN-CoVx. As the COVID-19 pandemic recently surfaced, a noticeable trend shows a “skewed dataset” for COVID-19 cases, though significantly, enough repositories are publicly available having radiography images of normal and viral or bacterial pneumonia-affected patients to compare to COVID-19-infection cases. Like ML models, deep-learning-based classification frameworks require a huge number of uniform data for proper model training to avoid challenges such as overfitting. In order to resolve such issues, this research employed various pre-processing techniques and a data-augmentation approach by exploiting stationary wavelets.

The course of the collection stage revealed that PeN-CoVx has images of various sizes; therefore, to maintain uniformity in size, each image was resized to 256 × 256 × 3 in the pre-processing stage. Before proceeding to data enhancement, the PeN-CoVx dataset was divided into training, validation, and testing sets with ratios of 60%, 25%, and 15%, respectively.

### 2.2. SW as Augmentation Approach

Besides using rotational and shear operation to extend the training set, this study exploited SW to perform data augmentation by decomposing images of training set up to three levels. Researchers in [20] introduced a non-sampling wavelet transformation called stationary wavelet transform with the capability of determining a more precise estimation of continuous wavelet transform by decamping a sample image into several levels. Besides this, it also possesses translational invariance and redundancy features.

The stationary wavelet decomposes an image based on the number of levels and the matrices’ dimensionality of the normalized input image. Assuming n as the number of decomposition levels, then SW transformation will produce 3-dimensional arrays for a 2-dimensional normalized matrix:

(1) $$C_{SW-2D}=\left[Ver\left(:,:,\;1:n\right);Hor\left(:,:,\;1:n\right);Dia\left(:,:,\;1:n\right);App\left(:,:,\;n\right)\right],$$

where *Ver* refers to the vertical coefficient; *Hor* represents the horizontal coefficient, and *Dia* denotes the diagonal coefficient, while *App* refers to the approximation coefficient for SW of *n*-levels. Similarly, for the 3-dimensional normalized image matrix (*h* × *w* × 3), then SW transformation will produce a coefficient with 4-dimensional arrays (*h* × *w* × 3 × *n*) for *n*-levels as in Equation (2) [20,21].

(2) $$C_{SW-3D}=\left[Ver\left(:,:,\;1:3,\;1:n\right);Hor\left(:,:,\;1:3,\;1:n\right);Dia\left(:,:,\;1:3,\;1:n\right);App\left(:,:,\;1:3,\;n\right)\right],$$

The present study exploited SW transformation for three levels of wavelet decomposition. Later, the decomposed images are randomly translated, rotated, and sheared to further enhance the PeN-CoVx training set. The experimental setup details about SW transformation and preprocessing steps can be found in the next section of this study.

### 2.3. Feature Generation Using Pre-Trained Frameworks

This section outlines the CNN-based TL models used to extract relevant features from the PeN-CoVx dataset.

#### 2.3.1. AlexNet

Back in 2012, Alex Krizhevsky and a handful of researchers presented a new landscape in the field of CV that demolished prior ideas by producing a seismic shift in resolving image classification tasks with AlexNet [22]. It was the winning entry in ILSVRC 2012 by solving an image classification problem for a dataset containing 1.4 million images of 1000 classes. AlexNet has deep architecture, composed of 650,000 neurons that contribute to 62.3 million training parameters. However, it incorporated dropouts [23] and ReLU instead of regularization and *tanh* to deal with issues such as overfitting and linearity, respectively. Moreover, it also utilized overlap pooling to reduce network size but introduced padding to prevent a drastic reduction in the size of the feature map.

It encompasses eight layers (three fully connected layers and five convolutional layers, CLs). The input layer in the network accepts an image with a size of 227 × 227. Next, the first CL has 96 filters with a size of 11 × 11 and stride 4, while the second CL has 256 filters but each with a size of 5 × 5 and stride 1. Furthermore, padding of 2 is utilized to handle the size of the feature maps. An overlapping max-pooling layer of 3 × 3 size with stride 2 follows each of these CLs. The rest of the CLs have 384 filters of size 3 × 3 with stride and padding of 1, except the last CL, which has 256 filters. The network extracts more features as the number of filters increases in deeper layers, and simultaneously, the feature map shape decreases as the filter size declines. It then has three fully connected layers, each having 4096 neurons except the last, which has 1000. A dropout of 0.5 is performed before and after the first fully connected layer to avoid overfitting.

#### 2.3.2. ResNet101

The prior deeper networks generally led to a degradation problem at the time of convergence, thus researchers suggested a residual network (ResNet) [24] to deal with such complications at ILSVRC 2015 competitions. Similarly, it outperformed other models while securing better generalization performance on COCO detection and segmentation in the Common Objects in Context (COCO 2015) competition. Prior to ResNet, deep learning researchers were developing deep networks by stacking more layers to extract complex features in order to attain high accuracy, but as the network converged, it faced a degradation problem. The increase in network depth, first, saturated the accuracy value, which then swiftly degraded due to the vanishing gradient effect. Reference [24] addressed the issue by introducing the ResNet framework, which stacks multiple CLs like any other deep network, but at the same time, it establishes identity connections between different layers. Besides numerous variants of ResNet, this study exploited ResNet101 because of its better performance on the problem at hand compared to the other variants described in the next section of this study. It mitigated the vanishing gradient effect and covariate shift problem through the incorporation of identity connection and batch normalization, respectively.

The ResNet101 architecture consists of four stages. It accepts an instance (input image), having a size of 224 × 224 × 3 in the case of this study, and passes it to the first CL (7 × 7 kernel) and then to the max- pooling (MP) layer with a kernel size of 3 × 3. The resultant is then fed to the first stage, which has three residual blocks with identity connections between them. Each block has three CLs of 64, 64, and 256 kernels, respectively. The second, third, and fourth stages have four, twenty-three, and three blocks, and each block has three CLs of different kernel sizes. After every network stage, the input size decline while the channel width doubles. Lastly, an average-pooling layer (AP) is placed, followed by a dense layer with a thousand neurons.

#### 2.3.3. SqueezeNet

In 2016, scientists at Stanford University and the University of California proposed a replacement for AlexNet, called SqueezeNet [25]. The devised compact network performs three times faster than AlexNet due to reduced parameters, as it replaces 3 × 3 filters with 1 × 1 filters to capture relationships among its channels. In addition, they used squeeze layers that reduces input channels to 3 × 3 filters. These strategies judiciously decrease the number of parameters in a CNN while pursuing the preservation of accuracy. Besides this, researchers introduced delayed down sampling in the network to attain large activation maps that maximizes the accuracy on a defined budget of parameters.

A fire module acts as a building block in the SqueezeNet architecture and is composed of *expand* and *squeeze* layers. The output of squeeze layers, each having a convolutional filter of size 1 × 1, is passed to the expand layer that contains 3 × 3 and 1 × 1 convolutional filters. The network contains eight fire modules, a global average-pooling layer, three max-pooling layers, and two standalone convolutional layers. Unlike other state-of-the-art networks, SqueezeNet does not have any dense layer.

The SqueezeNet has variants based on its architecture. Inspired by ResNet, the SqueezeNet architecture has several bypass connections to increase the filters. This study exploited SqueezeNet with a deep compression approach that uses 1 × 1 convolutional filters as the complex bypass connection designed in [25].

### 2.4. Feature-Selection Techniques

Feature selection is a crucial task in ML, as it has numerous options available but must choose useful features to design an efficient network. This study exploited three different feature-selection techniques, described below.

#### 2.4.1. Iterative Neighborhood Component Analysis (iNCA)

NCA is a technique to identify and segregate multivariate data by learning a distance metric [26]. It linearly transforms the original features to maximize classification performance. Functionally, it uses stochastic nearest neighbors (SNN) to differentiate the objective function by considering the whole transformed set instead of the k-nearest neighbors (kNN) at each transformed point for leave-one-out classification.

Instead of picking a fixed number of kNNs and taking a majority vote, it randomly picks a single neighbor point and determines the expected votes for each class. So each point *i* chooses another point *j* probabilistically by exploiting the softmax of the Euclidean distance between these two points using [26]:

(3) $$p_{ij}=\frac{\exp\left(-d_{i,j}\right)}{\sum_{k\neq i}\exp\left(-d_{i,k}\right)}\;,\;\;\;\;\;\;\;\;p_{i}=0,$$

where *d_i_*_,*j*_ refers to the Euclidean distance between a neighboring point and the LOO point. Thus, for better classification, NCA aims to maximize the objective function that can be defined as:

(4) $$f\left(A\right)=\sum_{i}\sum_{j\in S_{i}}p_{ij}=\underset{i}{\sum }p_{i},$$

where *S_i_* is the set of nearest neighbors.

#### 2.4.2. Iterative Chi-Square (iChi2)

Chi2 is a statistical technique used to analyze the dependency of events. Chi2 determines the deviation between the observed count, O, and the expected count, E, for data on two variables, using the following:

(5) $$x_{fr}^{2}=\sum^{\;}\frac{{\left(O_{i}+E_{i}\right)}^{2}}{E_{i}},$$

where *fr* refers to the degree of freedom. As in feature selection, the objective is to consider features that highly depend on the response, which can be achieved with a higher value of Chi2, if *O* is not close to *E*. Thus, a lower Chi2 value indicates that features are independent, while a higher value suggests that those representations are response-reliant and can be considered to train the model.

#### 2.4.3. Iterative Maximum Relevance–Minimum Redundancy (iMRMR)

Feature-selection algorithms can be classified into two groups, namely all relevant and minimal-optimal. Generally, all-relevant-based algorithms give individual assessment for each feature as they find statistical dependency with target variable. However, such techniques might be too indulgent in cases of many features with high redundancy. Therefore, ref. [27] discovered and proved that the iMRMR technique can achieve maximum accuracy even when selecting fewer features out of thousands for predicting a disease.

It works iteratively to identify an optimal set of features by calculating the iMRMR score for each feature using:

(6) $$x_{i}\left(f\right)=\frac{Rev(f\;|\;TV)}{Reu\;(f\;|\;features\;selected\;until\;i-1)},$$

where *f*, *Rev*, *Reu*, and *TV* refer to features under consideration, relevance, redundancy, and target variable, respectively. The feature that attains the highest score at the *i*th iteration is selected. In fact, it chooses a feature that has less redundancy with regard to features chosen at previous iteration, *i* − 1, but has higher relevance with regard to the target variable. Researchers suggested multiple variants of MRMR for discrete and continuous variables, but this study exploited a combine F-test with correlation using a quotient (FCQ) based on its popularity for various classification models in terms of robust accuracy and computational time.

### 2.5. Models for Classification

#### 2.5.1. Convolutional Neural Network

CNN is a widely used deep learning classification tool that has multiple perceptron layers placed next to each other in a sequence [28]. It is a chain of simple layers connected together to implement a form of progressive data distillation, by taking the input, transforming it to a meaningful representation, and predicting the final output. It usually encompasses three main forms of layers: convolutional, dense, and pooling layers. CL acts as a core component that consists of neurons in one layer allied with limited neurons of the neighboring layers, which shares weights having similar characteristics.

The relevant features are fed to CL (hidden layers) as an input feature map in a matrix or vector structure. The network determines a weight, *w*, for every connection between neurons of the CL and neurons from the first layer (input layer). According to first layer, the model then determines ‘*a*’ (weighted sums of all activations):

(7) $$w_{0,0}a_{0}^{\left(0\right)}+w_{0.1}a_{1}^{\left(0\right)}+\ldots \;w_{0,n}a_{n}^{\left(0\right)}$$

A function (such as σ sigmoid) is used to normalize the resultant, and the neuron activation is adjusted through the inclusion of appropriate bias, ‘b’, to represent the second neuron of the first hidden layer. In the same way, the network determines the weights and biases of every hidden layer to produce activation maps. Later, a pooling layer plays the role of a fuzzy filter by gradually downscaling the feature dimensionality to subsequently reduce computational cost. Lastly, a fully connected layer combines all the extracted representations of the preceding layers for classification.

#### 2.5.2. Linear Discriminant Analysis

Unlike logistic regression, the LDA traditional classification algorithm models differences in multi-classes by projecting data low dimensional-space. It determines the statistical properties from data to form a new axis of lower dimensional space that reduces intra-class variation while keeping greater differences in the means of classes. It calculates the mean value of each input *x* for every class:

(8) $$m_{z}=\frac{1}{n_{z}}\underset{i}{\sum }x_{i},\;$$

where *n_z_* is the number of instances within class *z*. It determines the variance across all classes using:

(9) $$v^{2}=\frac{1}{n-c}\underset{i}{\sum }{\left(x_{i}-m\right)}^{2},\;$$

where *c* equates to the number of classes. Lastly, it uses Bayes theorem to determine the probability for the new input set.

#### 2.5.3. Support Vector Machine (SVM)

SVM is widely adopted as a discriminative classifier to deal with linear as well as non-linear tasks. In order to correctly classify instances, it aims to define a decision boundary that maximizes the separating distance between the training data. Thus, it incorporates the first feature, known as the maximal margin classifier. It tries to find support vectors, instances located on the edge of class-descriptors, so that it can appropriately tag the samples into two classes. It is achieved by dividing the 2-dimensional space with a line such that data points falling on the left side of boundary are segregated into a different class than the ones on the other side. This division can be achieved with an infinite number of lines, but what makes SVM outstanding compared to the others (like k-nearest neighbors) is its ability to discover the best optimal possible separation line that has maximum margin between support vectors. This separating line is also known as a hyperplane if it is for a more-than-3-dimensional space by performing a dot product (kernel trick) on the transformed input vector.

A linear kernel is defined by the dot product of data *X* (which is to be categorized) with weight vector *w* (which the user wants to minimize) and the addition of a linearly estimated coefficient, *b*:

(10) $$Kernel\;Function\;\left(X\right)=w^{T}X+b.\;$$

The most used kernel for non-linear data is RBF, which computes distances between specific features with all others to generate new features. Therefore, this study used Gaussian RBF, which can be defined for *X*_1_ and *X*_2_ data by:

(11) $$Kernel\;Function\;\left(X_{1},\;X_{2}\right)=e^{\left(-\gamma *\;\|X_{1}-X_{2}\|{}^{2}\right)}.\;$$

## 3. Experimental Results and Analysis

The aim of this study is to design an efficient diagnostic tool that can accurately classify X-ray images of human lungs as COVID-19-infected, pneumonia or normal by enhancing a dataset using stationary wavelet transformation, generating relevant features via TL models, selecting optimal representations, and finally classifying using ML-based classifiers. In order to segregate the three best pre-trained TL models for feature generation, this study first tested several TL state-of-the-art networks with seven various ML and deep learning classifiers, tabulated in Table 1. Numerous experiments were conducted, wherein each TL network extracted useful features from images of the PeN-CoVx dataset; these images were later fed to the given classifiers for the diagnosis of the three clinical states. Table 1 puts forth the classification accuracies attained by CNN, DT, KNN, LDA, LR, NB, RF, and SVM against each pre-trained TL model on the PeN-CoVx dataset. Table 1 evidently shows that CNN, LDA, and SVM outperformed other classifiers (DT, KNN, LR, and RF) by effectively utilizing the features generated by TL networks. Similarly, AlexNet, ResNet101, and SqueezeNet successfully generated the most relevant features compared to other networks for the problem at hand, therefore; this research exploited AlexNet, ResNet101, and SqueezeNet as feature generators and CNN, LDA, and SVM for classification in the proposed scheme.

The PeN-CoVx dataset gathered for this research contained 4094 X-ray images for each clinical state (normal, COVID-19+, other pneumonia) and was further split into 60% training, 25% validation, and 15% testing sets, as listed in Table 2. As limited training dataset can lead to overfitting problem in TL-based models, this research employed various pre-processing techniques and data-augmentation approaches by exploiting SW transform, random rotation, translation, and shear operations. For this reason, the images are first normalized before decomposing them into various levels of stationary wavelets.

In the data-augmentation phase, the normalized PeN-CoVx images were decomposed to three levels using SWT. For this proposed study, each normalized image passed through several high-pass and low-pass filters. It exploited Daubechies’s (db2) orthogonal filter to decompose 2-D SW. For first-level decomposition, it determined the detail coefficients ($Ver_{1},\;Hor_{1},$ and $Dia_{1}$) and the approximate coefficient ($App_{1}$) of high and low frequencies, respectively. Similarly, for next-level decomposition, it determined the detail coefficients ($Ver_{2},\;Hor_{2},$ and $Dia_{2}$) and the approximate coefficient ($App_{2}$) of high and low frequencies, respectively. Likewise, the SWT data-augmentation phase produced three images for each corresponding image in the training set. Table 3 tabulates the detailed output coefficient for each level in 2-D SW.

In order to enhance the training dataset further, techniques including shear, random translation, and rotation were applied to each wavelet-decomposed image. Table 4 represents the parametric details of augmentation techniques exploited to overcome the overfitting problem. As a result, the number of images for each clinical state in training set was increased nine times; thus, the training data now contains 22,104 images for each class (normal, pneumonia, COVID-19+).

Later, three pre-trained TL models (described in the previous section) utilized these augmented training datasets to extract the 3000 most-relevant features for better classification. Before feeding images to the TL model, the size of the images was adjusted according to the input layer of each TL model. Each TL model (AlexNet, ResNet101, SqueezeNet) generated 1000 features for each image, which were then merged as shown in Algorithm 1. Thus, the merging accumulated 66,312 × 3000 features for all of the training sets.

**Algorithm 1.** Pseudo code of the proposed scheme.
**Proposed Algorithm**01*ImP = Load (PeN-CoVx)*  // Read PeN-CoVx image dataset02*ImNorm = (ImP – min(ImP)) / (max(ImP) – min(ImP))*  // Normalize images within range [0, 1]03*ImSWC = C_SW_(ImNorm)*  // Data augmentation: 3-levels decomposition by stationary wavelet transformation04*ImAug = DataAugmentation(ImSWC)*  // Performing random rotation, translation, and shear operation05for i = 1 to size-of *ImAug* do // Loop to extract relevant features for each image using transfer-learning model06   *fg_1_ = AlexNet(ImAug)*  // Extract 1000 features via AlexNet07   *fg_2_ = ResNet101(ImAug)*  // Extract 1000 features via ResNet10108   *fg_3_ = SqueezeNet(ImAug)*  // Extract 1000 features via SqueezeNet09   for j = 1 to 3 do // Loop to merge extracted feature10      *X(i, 1000 × j + 1: 1000 × (j + 1)) = fg_j_*  // merge extracted features11   end for loop // end loop to merge extracted feature12end for loop13*fs_1_ = iNCA(X, Y)*  // Determine optimal features via iterative Neighborhood Component Analysis14*fs_2_ = iChi2(X, Y)*  // Determine optimal features via iterative Chi-square15*fs_3_ = iMRMR(X, Y)*  // Determine optimal features via iterative Maximum Relevance Minimum Redundancy16*PL_k_ = CNN(fs_k_, Y, 3)*  // Predict clinical-state using optimal features by Convolutional Neural Network17*PL_k_ = LDA(fs_k_, Y, 3)*  // Predict clinical-state using optimal features by Linear Discriminant Analysis18*PL_k_ = SVM(fs_k_, Y, 3)*  // Predict clinical-state using optimal features by Support Vector Machine

Subsequently, the merged features were fed separately to three different feature-selection algorithms (iNCA, IChi2, and iMRMR). These feature selectors select optimal features to form vector by defining the upper and lower bound of the determined indices. Moreover, the feature-selection scheme iteratively calculates loss against each feature and finally selects features having low loss value as optimal features. Thus, iNCA, iChi2, and iMRMR successfully obtained 1588, 1620, and 1468 optimal features, respectively, that will contribute decisively in the classification of the three clinical states.

Lastly, one deep learning (CNN)- and two ML (LDA and SVM)-based classifiers were trained separately with 10-fold cross-validation for each optimal feature set obtained by iNCA, iChi2, and iMRMR. Later, each model was evaluated on an unseen test data set composed of 614 images of each class. As each classifier was trained separately for each iNCA feature vector, this study computed nine different classification schemes overall. For a better comparison, this study evaluated all trained models with the metrics accuracy, F1-score, recall, and precision.

After extensive trials, the most suitable CNN architecture was devised, having a few CLs and a fully connected layer. This CNN architecture contained three CLs with ReLU, stride of 1, and kernel size of 2. The first, second, and last CL had 256, 128, and 64 filter sizes, respectively. Averaging pooling followed the first and second CL for dimensionality reduction. Lastly, a fully connected layer with three neurons was utilized to classify an X-ray image into one of three-clinical states. Table 5 represents the classification performance achieved by CNN against each feature-selection technique.

Similarly, for each optimal feature set obtained from feature selectors, a separate LDA model was trained over 10-fold cross-validation. The model was trained by setting the gamma value as null with a linear discriminant type. Table 6 represents the classification performance achieved by LDA against each feature-selection technique.

Another ML classifier, SVM was separately trained over 10-fold cross-validation for each optimal feature set. RBF was used as a kernel function with auto scaling to classify the input X-ray image into three clinical states. Table 7 represents the classification performance achieved by SVM against each feature-selection technique.

## 4. Discussion

Besides analyzing the performance of the proposed scheme using accuracy, precision, sensitivity, and f1-score, we also determined the error of omission (see Table 8). This measures the false negatives of each classifier for all classes against each feature-selection technique to represent the fraction of samples belonging to a class but predicted to be in a different class. It is noteworthy that all proposed combinational schemes have less error of omission for COVID-19 cases than other classes (pneumonia and healthy individuals), except CNN with iNCA. The structural combination (CNN and iNCA) performed well for pneumonia classification, as it obtained only 0.81% error of omission. However, SVM with iChi2 outperformed all other schemes, as it has low error of omission on average for all classes.

Experts had developed numerous diagnostic tools to manage infectious diseases efficiently that can handle several class-classification tasks. For instance, ref. [29] blended conservative smoothing filtering, PCA, and SVM to secure 99.93% accuracy; however, it only segregated COVID-19-positive cases from negative cases. Contrarily, for a three-class classification task, ref. [30] used ResNet50 as the feature-extraction technique, with a SVM, to attain 95.33% accuracy. Similarly, ref. [31] exploited ResNet152 with various classifiers to reach an overall accuracy of 97.70% using X-ray images. Table 9 presents a comparative analysis of the proposed study with prior works.

This study suggested a robust smart diagnostic tool based on a modern TL framework and iterative feature-selector techniques to rapidly screen and accurately diagnose COVID-19, healthy individuals and other pneumonia using radiography images. It tested various TL networks and finally selected three TL architectures (AlexNet, ResNet101, and SqueezeNet) to generate the most-relevant features from radiography images. Later, it employed three feature-selection techniques (iNCA, iChi2, and iMRMR) and analyzed their effects on the proposed deep-learning-based CNN framework, LDA, and SVM classifiers to classify COVID-19+, other pneumonia, and healthy cases.

For this experimental task, 12,282 radiography images were examined, of which 4094 belonged to COVID-19 infected patients and 4094 belonged to other pneumonia cases, while the rest pertained to normal patients or healthy humans. Extensive experiments were performed to ensure that the proposed model scheme generalized well using 10-fold cross-validation to cater to unseen data and does not perform overfitting or underfitting. By practicing feature generators (AlexNet, ResNet101, and SqueezeNet) along iChi2 with SVM, the network achieved an overall accuracy of 99.2% with minimal computational time. Likewise, combinations of feature generators with iChi2 and CNN also achieved 99.0%. Evidently, the proposed scheme ((AlexNet, ResNet101, SqueezeNet) + iChi2 + SVM) surpassed prior studies.

Contrary to previously well-established studies that utilized fewer samples of COVID-19 cases for training, this proposed study analyzed 4094 X-ray images labeled as COVID-19 positive and also practiced stationary-wavelet-transform-based data augmentation and the k-fold validation method to prevent overfitting. Undoubtedly, prior studies adopted pre-trained networks as a base classifier without implementing any feature generator and selector technique together. In contrast, this work is composed of various TL-based feature generators and several iterative feature-selector techniques that eliminates redundant features and significantly affects the accuracy of the proposed diagnosis decision-making system. Unlike previous studies, the experimental work of this study showed that selecting a relatively shallow network even produced optimal results with less computational time when trained with an augmented data set. Such schemes can be exploited for tasks discussed in [39,40] to manage health services more efficiently.

## 5. Conclusions

The recent outbreak of coronavirus disease has severely affected more than 560 million individuals around the globe, causing more than 6.3 million deaths to date. Besides smart diagnostic tools, presently, the only standard practiced to identify COVID-19 positive cases is by taking samples of the respiratory tract through viral nucleic acid testing. In this paper, we investigated a framework for COVID-19 and other viral/bacterial pneumonia identification from X-ray images by employing a combination of TL models (AlexNet, ResNet101, and SqueezeNet) as a feature generator, feature selectors (iNCA, iChi2, iMRMR), and ML classifiers. The TL networks were helpful for generating the relevant features from X-ray images; the features were refined by applying a feature-selection approach using iNCA, iChi2, and iMRMR. The feature-selection process not only helped identify highly discriminative features, but it also reduced the processing speed for training the classifiers by removing unwanted or redundant features. Finally, CNN, LDA, and SVM were investigated for the detection of COVID- 19-positive cases among viral/bacterial pneumonia and normal cases. In addition to this, the study exploited stationary wavelet transform to enhance a limited training dataset by decomposing each training image up to three levels. Furthermore, it also incorporated random rotation, translation, and shear to augment the data set by a factor of nine. The results presented in this study indicate that the proposed scheme can be used as a smart diagnostic tool for the identification and monitoring of COVID-19 cases, as well as other pneumonia cases, as it achieved an overall accuracy of 99.24%. The results indicated that using a combination of computer vision and deep learning techniques on X-ray images of lungs can help with the identification of COVID-19 cases to reduce the burden on healthcare workers in the time of the pandemic. In the future, the integration of clinical results along with radiography imaging can be exploited for the better detection and diagnosis of COVID-19-positive cases, with the addition of tuberculosis and lung cancer.

## Figures and Tables

**Table 1:** Comparison analysis of pre-trained transferred models as feature-extractor and machine-learning-classification algorithms.

Feature-Generation Models	Classification Accuracy (%)						
	CNN	DT	KNN	LDA	LR	RF	SVM
AlexNet	95.6	90.7	88.8	95.0	91.9	90.4	**95.9**
DenseNet-121	95.0	90.4	87.5	94.8	91.9	91.0	94.8
DenseNet-169	95.1	91.0	89.5	95.1	92.0	91.0	95.3
DenseNet-201	95.1	90.1	87.6	94.5	90.7	88.9	95.3
DenseNet-263	94.8	88.8	85.0	93.8	90.1	87.5	95.0
EfficientNet-B0	92.9	86.6	8.9	90.7	86.6	86.8	91.5
GoogleNet	93.5	90.1	82.2	92.5	87.1	88.8	92.5
InceptionNetV3	95.0	91.0	83.1	93.3	87.3	89.3	95.1
MobileNetV2	90.2	87.5	80.1	89.9	85.0	87.5	90.1
ResNet18	90.7	88.8	82.2	90.7	86.7	85.0	90.6
ResNet50	92.5	86.6	82.6	91.9	87.1	85.5	92.9
ResNet101	**96.1**	91.9	85.0	95.9	90.7	87.8	**96.1**
ResNet152	95.1	91.2	85.3	95.0	90.7	90.4	95.3
SqueezeNet	96.1	93.5	85.0	96.1	91.5	92.2	**96.6**
VGG16	93.5	90.1	84.4	91.9	88.6	91.9	93.0
VGG19	92.9	90.7	83.1	92.9	88.6	91.5	92.5
XceptionNet	91.0	87.5	80.5	88.8	87.5	86.8	91.0

CNN: convolutional neural network; DT: decision tree; KNN: k-nearest neighbors; LDA: linear discriminant analysis; LR: logistic regression; RF: random forest; SVM: support vector machine.

**Table 2:** Data set information.

Clinical State	Number of Instances			
	Training Set without Augmentation	Training Set with Augmentation	Validation Set	Testing Set
Normal	2456	22,104	1024	614
Other pneumonia	2456	22,104	1024	614
COVID-19+	2456	22,104	1024	614
**Total**	**7368**	**66,312**	**3072**	**1842**

**Table 3:** Output coefficient for each level in 2-D stationary wavelet (SW) transform.

Decomposition Level	Down Sampling	Approximate Coefficient (Low Frequency)	Detail Coefficient (High Frequency)
1	Yes (by 2)	*App*_1_	*Ver*_1_, *Hor*_1_, *Dia*_1_
2	-	*App*_2_	*Ver*_2_, *Hor*_2_, *Dia*_2_
3	-	*App*_3_	*Ver*_3_, *Hor*_3_, *Dia*_3_

*App*: approximate coefficient (image); *Ver*: vertical coefficient; *Hor*: horizontal coefficient; *Dia*: diagonal coefficient of the SW transformed image.

**Table 4:** Parametric values of data-augmentation techniques.

Augmentation Technique	Parametric
Translation	−10, 10
Rotation	−90, 90
Shear	−30, 30

**Table 5:** Performance metrics for the convolutional neural network against each feature-selection technique.

Feature Selector	Statistics	Precision	Recall	F1-Score	Accuracy
iNCA	Minimum	98.536	98.534	98.535	98.534
	Maximum	99.078	99.077	99.077	99.077
	Average	98.810	98.806	98.808	98.806
**iChi2**	Minimum	98.915	98.914	98.914	98.914
	Maximum	99.133	99.132	99.132	99.131
	**Average**	**99.026**	**99.023**	**99.024**	**99.023**
iMRMR	Minimum	98.489	98.154	98.322	98.154
	Maximum	98.914	98.914	98.914	98.914
	Average	98.535	98.534	98.534	98.534

iNCA: iterative neighborhood component analysis; iChi2: iterative chi-square; iMRMR: iterative maximum relevance–minimum redundancy.

**Table 6:** Performance metrics for linear discriminant analysis against each feature-selection technique.

Feature Selector	Statistics	Precision	Recall	F1-Score	Accuracy
**iNCA**	Minimum	97.790	97.768	97.779	97.774
	Maximum	98.752	98.751	98.751	98.751
	**Average**	**98.271**	**98.263**	**98.267**	**98.263**
iChi2	Minimum	97.674	97.666	97.670	97.666
	Maximum	98.207	98.209	98.208	98.208
	Average	97.535	97.515	97.525	97.515
iMRMR	Minimum	96.526	96.526	96.526	96.526
	Maximum	97.927	97.720	97.724	97.720
	Average	97.124	97.123	97.123	97.123

iNCA: iterative neighborhood component analysis; iChi2: iterative chi-square; iMRMR: iterative maximum relevance–minimum redundancy.

**Table 7:** Performance metrics for the support vector machine against each feature-selection technique.

Feature Selector	Statistics	Precision	Recall	F1-Score	Accuracy
iNCA	Minimum	98.756	98.751	98.754	98.751
	Maximum	99.191	99.186	99.189	99.186
	Average	98.969	98.969	98.969	98.969
**iChi2**	Minimum	99.024	99.023	99.023	99.023
	Maximum	99.462	99.457	99.460	99.457
	**Average**	**99.241**	**99.240**	**99.241**	**99.240**
iMRMR	Minimum	98.534	98.534	98.534	98.534
	Maximum	99.078	99.077	99.078	99.077
	Average	98.806	98.806	98.806	98.806

iNCA: iterative neighborhood component analysis; iChi2: iterative chi-square; iMRMR: iterative maximum relevance–minimum redundancy.

**Table 8:** Comparing the error of omission of the convolutional neural network (CNN), linear discriminant analysis (LDA), and support vector machine (SVM) for each class against the exploited feature-selection techniques.

Feature Selector	Class Label	Error of Omission (%)		
		CNN	LDA	SVM
iNCA	Normal	1.63	2.12	1.14
	Pneumonia	0.81	1.63	1.14
	COVID-19	1.14	1.47	0.81
iChi2	Normal	1.14	2.12	0.98
	Pneumonia	0.98	2.44	0.65
	COVID-19	0.81	1.63	0.65
iMRMR	Normal	1.47	3.09	1.30
	Pneumonia	1.79	3.26	1.30
	COVID-19	1.14	2.28	0.98

iNCA: iterative neighborhood component analysis; iChi2: iterative chi-square; iMRMR: iterative maximum relevance–minimum redundancy.

**Table 9:** Comparative analysis of the proposed study with prior works.

Study	Techniques	Accuracy (%)
[30]	ResNet50 feature extractor with SVM	95.33
[31]	SMOTE and ResNet152 with XGBoost and random forest	97.70
[32]	Customized CNN-based network	84.22
[33]	VGG-16-based scheme	97.0
[34]	Customized Xception Net	95.0
[35]	CNN with transfer multireceptive feature optimizer	95.1
[36]	Cascaded ResNet50V2 and Xception Net	91.4
[37]	Customized CNN-based model	93.30
[38]	Pre-trained deep learning models with GAN	85.2
**Proposed**	**SWT + (AlexNet, ResNet101, and SqueezeNet) + iChi2 + SVM**	**99.24**

SVM: support vector machine; CNN: convolutional neural network; GAN generative adversarial network; SWT: stationary wavelet transform; iChi2: iterative chi-square.

## Data Availability

No new data is generated for this study.
